# Neural Network Interpretation of the Intensity of Damage Processes to Biological Membranes of Human Cells, Depending on the Degree of Polymetallic Contamination of the Territory

**DOI:** 10.3390/biomedicines14061190

**Published:** 2026-05-25

**Authors:** Yulia A. Tunakova, Svetlana V. Novikova, Vsevolod S. Valiev

**Affiliations:** 1 Department of General Chemistry and Ecology, Kazan National Research Technical University Named After A. N. Tupolev—KAI, Kazan 420111, Russia; yuatunakova@kai.ru; 2 Department of Applied Mathematics and Computer Science and Research Laboratory Environmental and Industrial Safety, Kazan National Research Technical University Named After A. N. Tupolev—KAI, 10 K. Marx St., Kazan 420111, Russia; 3 Research Institute for Problems of Ecology and Mineral Wealth Use of Tatarstan Academy of Sciences, 28 Daurskaya St., Kazan 420087, Russia; ipen-anrt@mail.ru

**Keywords:** biosensing, malondialdehyde detection, oxidative stress biomarkers, biomarker-based modeling, environmental health sensing, neural network modeling, children’s health monitoring

## Abstract

**Background:** Lipid peroxidation is a primary driver of biological membrane damage and mediates the relationship between environmental exposure and adverse health outcomes. Malondialdehyde (MDA) is a widely recognized biomarker for quantifying oxidative stress intensity. Despite numerous studies on oxidative stress and metal exposure, nonlinear relationships between physiological characteristics, serum metal profiles and MDA levels in pubertal children remain insufficiently studied. **Methods:** The study included 105 conditionally healthy children aged 12–14 years from urban and rural regions of Tatarstan, Russia. Serum MDA concentrations were determined spectrophotometrically using the thiobarbituric acid assay, while Zn, Cu, Fe, Sr and Pb concentrations were measured by atomic absorption spectrometry. A multilayer perceptron neural network was applied to model nonlinear relationships between MDA levels, environmental exposure indicators and morphophysiological characteristics. Because the original relational dataset contained partially replicated participant-derived relational structures, primary validation was performed using independently reconstructed datasets without repeated observations. Additional repeated cross-validation and SHAP-based feature importance analysis were performed. **Results:** Urban-residing children demonstrated significantly higher serum MDA levels than rural counterparts, independent of sex, with girls consistently showing higher values. Reduction of predictor dimensionality improved model generalization behaviour. Validation using independently reconstructed datasets without repeated observations demonstrated reproducible exploratory predictive behaviour of the reduced neural network model, with independently reconstructed validation datasets yielding mean R^2^ values of 0.901 ± 0.052 and 0.914 ± 0.046, respectively. SHAP analysis demonstrated that zinc, copper and iron consistently represented the dominant contributors to the nonlinear model, although substantial variability in the relative ranking of zinc and copper was observed between validation datasets. **Conclusions:** The proposed neural network model demonstrated the ability to capture reproducible nonlinear relationships between oxidative stress markers and environmental exposure parameters in a limited biomedical dataset. The model should primarily be interpreted as an exploratory explanatory tool rather than an individual clinical prediction instrument. Because of the limited dataset size, partially reconstructed relational structure and exploratory study design, the findings require cautious interpretation and further external validation.

## 1. Introduction

Cell damage mechanisms in organs and systems originate at the level of biological membranes. Key mechanisms involve the disruption of membrane integrity by reactive oxygen species (ROS) and the imbalance between antioxidant defenses and ROS production. This imbalance leads to the oxidation of unsaturated fatty acids in cell membranes, producing lipid peroxides and toxic oxidation products that compromise cell viability. The sequence of lipid peroxidation (LPO) reactions is a central mechanism damaging biological membranes and contributes significantly to disease development [1,2]. LPO involvement has been documented in diseases such as atherosclerosis, circulatory disorders, diabetes mellitus, rheumatoid arthritis, inflammatory and degenerative disorders, cancers, burns, intoxications, and premature aging [3,4,5,6,7,8,9,10,11].

Oxidation products of the lipid layer, entering the blood plasma, can form elevated levels that are dynamically assessed [4,5,12]. As a result, blood is regarded as the most important object of study, serving as a sensitive indicator that reflects the state of individual systems and the organism as a whole.

Malondialdehyde (MDA) is one of the end products of lipid peroxidation. Experimentally, lipid peroxidation is most often assessed by measuring MDA levels in blood serum or plasma [12,13,14,15,16]. Assessment of serum MDA levels is informative for diagnosing a wide range of diseases and conditions [17].

Metals with variable valence can initiate a variety of radical-forming reactions. As a result of free radical action, membrane lipids undergo oxidative degradation, disrupting membrane integrity. Because metals play a significant role in regulating various stages of the free-radical chain oxidation reaction, the accumulation of LPO products in the human body is associated with the levels of several metals (Fe, Cu, Zn, etc.) in biosubstrates [18,19]. It is these metals (Fe, Cu, and Zn) that show the closest association with MDA levels: Fe induces Fenton reactions, Cu is a component of serum ceruloplasmin, and both Cu and Zn act as cofactors of superoxide dismutase (CuZnSOD or SOD1). These conclusions are supported by a number of recent publications [20,21]. Notably, the intensity of LPO processes increases under conditions of polymetallic environmental pollution [22,23,24,25].

Confirmation of this postulate is provided by studies assessing the levels of several metals in blood, hair, and drinking water in children, as well as quantitative determinations of MDA levels in Brazil. The results demonstrated increased serum MDA levels, elevated blood lead and nickel concentrations, and elevated aluminum levels in hair and drinking water among children from polluted areas [26,27].

A study by Japanese researchers reported results from an assessment of the relationship between serum zinc and MDA in 100 children (aged 6 months to 5 years), of whom 50 had malnutrition, and 50 served as a control group. Notably, MDA concentrations were significantly higher in malnourished children compared with the control group [28], confirming the influence of body weight on this process.

It has been established that environmental pollution in schoolchildren aged 8–13 years in China with metals (waste from electronic equipment processing) leads to increased concentrations of these metals (in particular, manganese and nickel) in blood serum, accompanied by a parallel increase in MDA levels. This effect is especially pronounced at ages 8–9 years [29]. Similar findings were observed in children aged 3–6 years exposed to lead [30].

In 12-year-old schoolchildren from the industrial center of Lucknow, India, elevated blood lead levels were observed, which correlated with serum MDA levels (r = 0.46, *p* = 0.00018). It was concluded that MDA values, together with a number of other biochemical parameters, can be used as a biomarker of lead intoxication [31]. Approximate serum MDA levels in children are presented in [32].

Despite numerous studies investigating oxidative stress biomarkers and environmental metal exposure, nonlinear relationships between physiological characteristics, serum metal profiles and lipid peroxidation markers in pubertal children remain insufficiently studied. In particular, the application of interpretable machine learning approaches for exploratory modelling of oxidative stress biomarkers in environmentally exposed pediatric populations remains limited.

The aim of the study was to investigate the relationship between environmental metal exposure and lipid peroxidation intensity and to evaluate the ability of a neural network model to describe nonlinear dependencies between physiological parameters and serum MDA levels in a limited biomedical dataset.

## 2. Materials and Methods

### 2.1. Initial Data

The object of the study was blood serum from potentially healthy children (with no chronic diseases at the time of examination) aged 12–14 years during puberty, living in areas with different levels of anthropogenic load of the urban environment, as a target and the most sensitive part of the population. Information regarding the absence of chronic diseases and acute infectious conditions at the time of examination was obtained from available medical documentation and participant health questionnaires collected during the study. A total of 105 children participated in the study: 72 from the city of Kazan (a territory with a high level of polymetallic pollution) and 33 from rural areas (the Vysokogorsky and Arsky districts of the Republic of Tatarstan), considered conditionally unpolluted territories. The classification of urban and rural territories was based on official regional environmental monitoring data reported in the State Report on the State of Natural Resources and Environmental Protection of the Republic of Tatarstan (Ministry of Ecology of the Republic of Tatarstan) [33].

Serum samples were collected strictly under fasting conditions (8–12 h of fasting) in the morning from the antecubital vein. The exploratory relational dataset contained 200 records derived from data obtained from 105 biological participants. The relational modelling framework was used to analyse nonlinear associations between demographic, physiological, environmental and biochemical variables within the machine learning pipeline. The framework retained alternative relational representations of the same participant within different exploratory modelling contexts used during preliminary model construction and comparison procedures. In this framework, several analytical representations associated with the same participant could be retained during exploratory modelling procedures, resulting in partial relational replication within the dataset structure. Consequently, some relational rows represented partially replicated participant-derived structures and therefore should not be interpreted as fully independent biological observations. No synthetic biological values were generated, and no artificial augmentation of measured biochemical parameters was performed. Because such relational structures may introduce statistical dependencies between rows, additional leakage-aware validation using independently reconstructed datasets without repeated observations was subsequently performed.

All analysed biological and clinical data were fully anonymized prior to machine learning analysis, and no personally identifiable participant information was available to the investigators during data processing. According to the decision of the Local Ethics Committee, separate written informed consent for the analytical use of anonymized data was waived within the approved study framework.

In the present study, a generally accepted method for determining MDA concentration based on its reaction with thiobarbituric acid (TBA) was used [34,35]. TBA was dissolved in the presence of Triton X-100 to prevent its precipitation. To stabilize the trimethine complex, Trilon B was added, and denatured serum proteins were dissolved in a mixture of ethanol and chloroform (7:3), as described by [36].

At high temperature and in an acidic medium, the reaction between MDA and TBA proceeds with the formation of a pink-colored trimethine complex containing one MDA molecule and two TBA molecules. The absorption maximum of the complex is at 532 nm.

The working TBA solution was prepared by dissolving 864 mg of TBA in 100 mL of a mixture containing 1% Triton X-100 and 8.2 M ethanol. All other solutions were prepared using bidistilled water.

To 1.5 mL of serum, 0.5 mL of a 1% Triton X-100 solution, 0.2 mL of a 0.6 M HCl solution, and 0.8 mL of a 0.06 M working TBA solution were added sequentially. The mixture was heated in a boiling water bath for 10 min and then cooled to 15 °C for 30 min. To stabilize the color, 0.2 mL of a 5 mM Trilon B solution was added, and the volume was adjusted to 10 mL with a mixture of ethanol and chloroform (7:3). Optical density was measured at 532 nm using an SF-46 spectrophotometer in a 1 cm glass cuvette. A blank sample, in which bidistilled water was added instead of serum, served as the control. To minimize matrix interference, additional blank samples subjected to the complete protein precipitation procedure were analysed. Purity of the absorption peak at 532 nm was verified by spectrophotometric scanning and absence of secondary shoulders in the 550–560 nm region characteristic of nonspecific sugar-related reactions. In the MDA calculations, a molar extinction coefficient of 0.156 µM^−1^·cm^−1^ was used.

The concentration of malondialdehyde in blood serum was calculated using the formula:

(1) $$C_{MDA}=\frac{\left(D_{1}-D_{2}\right)\times U_{2}}{\epsilon \;\times L\;\times U_{1}}$$

where *D*_1_ is the optical density of the serum sample; *D*_2_ is the optical density of the control; *U*_1_ is the volume of serum taken for analysis (1.5 mL); *U*_2_ is the final volume of the mixture (10 mL); L is the cuvette path length (1 cm); and ε = 0.156 is the molar extinction coefficient of the MDA–TBA complex (*L*·µmol^−1^·cm^−1^). The result is expressed in µmol/L.

In the obtained blood serum, the contents of iron (Fe), copper (Cu), zinc (Zn), strontium (Sr), and lead (Pb) were determined by atomic absorption spectrometry using an AAnalyst 400 instrument (PerkinElmer, Shelton, CT, USA).

Zinc was determined at the resonance line of 213.9 nm with a detection limit of 1.5 µg/L; copper at 324.8 nm with a detection limit of 1 µg/L; iron at 248.3 nm with a detection limit of 5 µg/L; strontium at 460.7 nm with a detection limit of 3 µg/L; and lead at 283.3 nm with a detection limit of 7 µg/L. Lead was determined using an electrodeless high-frequency lamp, while the other metals were measured with hollow-cathode lamps.

Calibration solutions were prepared by appropriate dilution of certified reference materials. Formal LOD and LOQ values for the TBA-based MDA assay were not separately estimated because the method was applied in a comparative mode aimed at identifying relative intergroup differences rather than absolute analytical quantification. The concentrations of Zn, Cu, Fe, and Sr were measured directly in blood serum after dilution at a ratio of 1:2 with bidistilled water. Lead determination was performed after protein precipitation. For this purpose, 0.75 mL of a 1.5% HCl solution was added to 1.5 mL of blood serum, and the mixture was incubated for 1 h at 37 °C. After protein hydrolysis, proteins were precipitated with 0.75 mL of 20% trichloroacetic acid (TCA) and centrifuged for 10 min at 1500 rpm; the dilution ratio was also 1:2. The supernatant was used for analysis. The result is expressed in mg/L. Because serum samples were analysed, the measured metal concentrations primarily reflect circulating mobile fractions associated with transport proteins rather than total body metal burden.

Since the dependence of MDA on the eleven identified predictors is markedly nonlinear, it is appropriate to use machine learning methods to model MDA concentration. In particular, multilayer perceptrons have proven effective in similar tasks, serving as universal approximators [37]. Similar exploratory applications of machine learning methods for oxidative stress and environmental exposure assessment have been reported in recent biomedical studies; however, most previous works focused primarily on predictive performance rather than interpretability of nonlinear biological relationships. In the present study, additional SHAP analysis was incorporated to improve transparency of the neural network model and reduce its “black-box” character.

### 2.2. Model

We built the model using relational data formatted as tables. Rows represent tuples, and columns represent attributes. The initial relational dataset contained 200 relational rows constructed from 105 biological participants within an exploratory relational modelling framework. The data were divided into training and testing sets in proportions of 95% and 5%, or 190 and 10 tuples. The hold-out subset was used exclusively for preliminary overfitting detection and exploratory comparison of model behaviour rather than for estimation of final predictive performance. Final robustness-oriented validation results are reported using independently reconstructed datasets without repeated observations.

### 2.3. Data Preprocessing

Prior to modelling, categorical variables (sex and place of residence) were converted into numerical format using binary encoding. Continuous variables were linearly normalized to the range [−1, 1]. The dataset did not contain missing values. Outliers were not removed, as all measurements were considered physiologically plausible and representative of real biological variability. All preprocessing procedures were performed after splitting the dataset into training and test subsets to prevent data leakage.

### 2.4. Model Validation and Overfitting Control

To evaluate model reliability for a limited biomedical dataset, additional generalization control procedures were applied. The ratio between model complexity and dataset size was assessed by comparing training and test errors and analysing bias–variance behaviour. Overfitting was assumed when training accuracy significantly exceeded test accuracy. Predictor reduction was performed using correlation analysis and multicollinearity diagnostics to reduce model capacity and improve generalization.

To improve validation reliability for the reduced neural network model, repeated cross-validation was additionally performed. The validation procedure included repeated 5-fold cross-validation with 20 repetitions using different random partitions of the dataset (100 validation runs in total). For each iteration, the Mean Absolute Error (MAE) and coefficient of determination (R^2^) were calculated.

Because the relational dataset contained partially replicated participant-derived relational structures, additional robustness-oriented validation procedures were performed. Two independently reconstructed datasets without repeated observations were generated from the original dataset. In these supplementary datasets, duplicated or partially replicated relational structures were removed, ensuring that each retained observation corresponded to a single participant representation. Repeated 5-fold cross-validation was then independently applied to each reconstructed dataset in order to evaluate reproducibility and stability of model performance under stricter independence conditions and to reduce the risk of intra-patient information leakage during model validation. Each reconstructed dataset contained only one retained relational representation per participant. This procedure was intended to reduce the probability of intra-participant information leakage during model validation.

The extended robustness-oriented validation procedures were primarily focused on the reduced neural network model because the reduced architecture demonstrated improved generalization ability and lower overfitting compared with the full model.

### 2.5. Training Procedure

The network was trained using backpropagation with mean squared error (MSE) as the loss function. A fixed learning configuration was used without dedicated hyperparameter optimization in order to isolate the effect of predictor dimensionality. Regularization techniques (e.g., dropout) were not applied because overfitting control was achieved through reduction of model capacity. Training was performed for a fixed number of epochs until convergence of the training error. The learning rate was selected empirically using the default configuration of the applied neural network implementation environment.

## 3. Results

### 3.1. Descriptive Statistics and Group Differences

The distribution of the obtained MDA values and metal concentrations was statistically assessed (Statistica 6): mean values (M) and standard deviations (SD) were calculated for the study groups; the statistical significance of differences was assessed using the Mann–Whitney U test; and correlations were assessed using the Pearson method. Discriminant analysis and multiple regression were used to assess the significance of individual parameters.

The obtained results for MDA and metal levels in serum, distributed by study sites and groups, are presented in Table 1.

When evaluating the results in Table 1, note that higher MDA concentrations were observed in children living in areas with a high degree of anthropogenic load, regardless of sex. Girls showed higher MDA levels despite similar chronological age, which may reflect differences in pubertal maturation. A positive correlation between MDA and body surface area was observed, suggesting increased metabolic activity. Earlier hormonal maturation in females during puberty may enhance oxidative processes and lipid peroxidation.

To calculate serum MDA concentrations, heterogeneous datasets were used, accounting not only for the metal content in blood serum but also for subjects’ physiological characteristics (sex, height, weight, body surface area, and age), as well as the degree of anthropogenic load. Based on the above, in order to model the dependence of serum MDA concentrations on physiological and environmental parameters, it was decided to construct the model using the following set of predictors:

1. Blood metal concentrations (mg/L): Zn, Fe, Cu, Sr, Pb.
2. Physiological characteristics: sex; age (years); body weight (kg); height (cm); body surface area (m^2^).
3. Characteristics of the area of residence: rural area/urban area.

The study does not isolate individual environmental determinants such as particulate matter exposure or diet. Instead, the residential area represents an integrated exposure proxy reflecting the combined long-term influence of environmental and lifestyle factors. Thus, the results should be interpreted as effects of cumulative environmental load rather than specific pollutants.

The applied urban-versus-rural categorization was intentionally used as a simplified exposure framework to identify potential long-term differences associated with industrial and transport-related anthropogenic load.

### 3.2. Neural Network Model Using the Full Predictor Set

**Figure 1:**
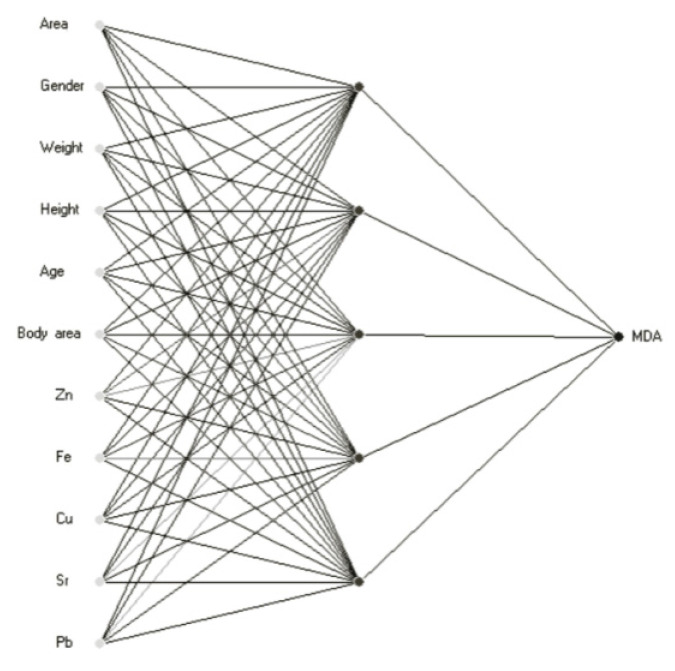
Architecture of the neural network model for calculating MDA using a full set of predictors.

Based on the available dataset, a neural network model with the following structure was designed (Figure 1):

- Number of input neurons: 11;
- Number of hidden layers: 1;
- Number of neurons in the hidden layer: 5;
- Number of output neurons: 1;
- Activation function of the hidden layer neurons: hyperbolic tangent;
- Activation function of the output layer: linear.

Input variables were normalized to the range [−1, 1] using linear scaling due to their heterogeneity.

Analysis of the modeling results revealed the following. Within the preliminary exploratory hold-out configuration based on the original relational dataset, the model demonstrated high apparent training accuracy (R^2^ = 0.95), while test performance decreased substantially, indicating pronounced overfitting and limited generalization ability. However, on the test data, the error increased fivefold, while the R^2^ coefficient of determination decreased by more than half.

**Figure 2:**
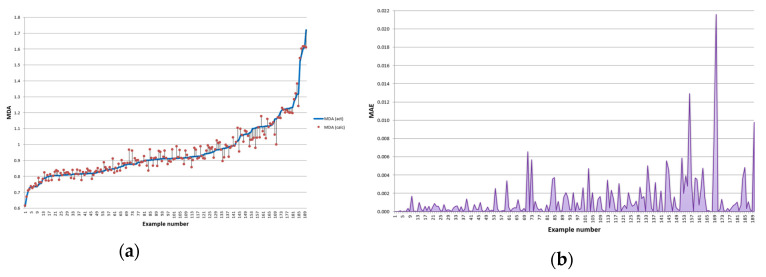
Deviation of calculated (calc) and experimental (act) values of MDA on the training sample for the full set of predictors: (**a**) comparison of values; (**b**) value of absolute error for training examples.

**Figure 3:**
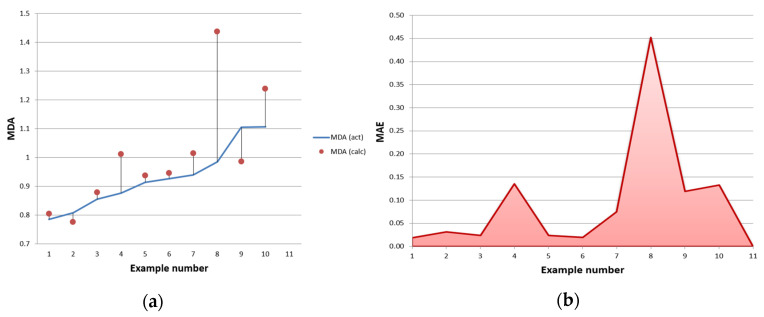
Deviation of calculated (calc) and experimental (act) values of MDA on the test sample for the full set of predictors: (**a**) comparison of values; (**b**) value of absolute error for test examples.

Figure 2 and Figure 3 illustrate the deviation of the model-calculated MDA values from the experimental ones for different samples.

### 3.3. Reduced Neural Network Model

Analysis of the histograms of the observed MDA values and those calculated using the constructed model also revealed a mismatch between the training and test data. While the distributions of experimental and predicted values across ranges coincided in most cases in the training set, in the test data, a significant shift toward higher MDA values was observed. Such results indicate model overfitting and poor generalization.

Obviously, reducing the number of input neurons simplifies the neural network structure and, as a result, improves its generalization ability. Because the dataset is limited, different splitting strategies (including deterministic sampling methods) may slightly change numerical error values but do not affect the qualitative conclusion regarding overfitting and generalization behaviour.

Additional correlation analysis of the input parameters revealed that three predictors: “Age,” “Height,” and “Weight” had a weak influence on the output or were multicollinear. The dataset was first divided into training and test subsets. Feature selection (correlation analysis and multicollinearity assessment) was performed using only the training data. The resulting predictor set was then fixed and applied unchanged to the test subset to avoid data leakage.

The reduced neural network model with the eight most significant predictors has an architecture similar to that of the full model, differing only in the number of input neurons:

- Number of input neurons: 8;
- Number of hidden layers: 1;
- Number of neurons in the hidden layer: 5;
- Number of output neurons: 1;
- Activation function of the hidden layer neurons: hyperbolic tangent;
- Activation function of the output layer: linear.

The modeling results showed significant convergence in model performance across the training and test sets, the test error decreased approximately twofold, suggesting improved generalization behaviour. The following hold-out results are presented only as preliminary exploratory estimates obtained from the original relational dataset and should not be interpreted as leakage-free validation metrics (Table 2).

The slightly higher test-set R^2^ value likely reflects random variability associated with the very small exploratory hold-out subset and should not be interpreted as evidence of superior generalization. The convergence of performance metrics between training and test sets suggests reduced memorization effects and suggests that the dataset size appears relatively adequate for the reduced network architecture. In contrast, the full model demonstrated high variance typical for over-parameterized models trained on limited datasets. Therefore, predictor reduction improved generalization stability of the model.

The modelling error (MAE = 0.051 µmol/L) corresponds to approximately 4–5% of the mean MDA level and is considerably smaller than the biological differences observed between exposure groups. Therefore, the model accuracy is sufficient for population-level environmental monitoring rather than individual clinical diagnostics.

**Figure 4:**
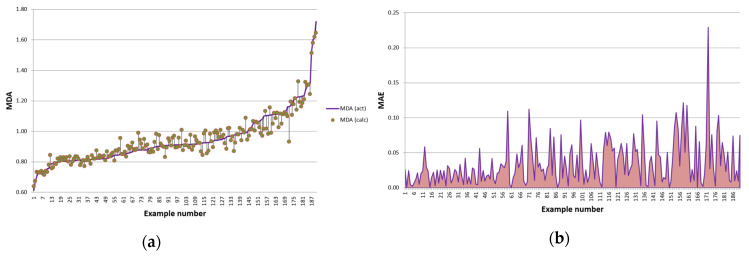
Deviation of calculated (calc) and experimental (act) values of MDA on the training sample for a reduced set of predictors: (**a**) comparison of values; (**b**) value of absolute error for training examples.

**Figure 5:**
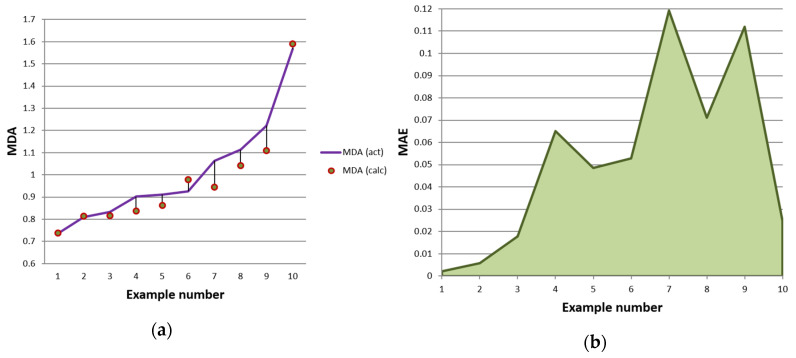
Deviation of calculated (calc) and experimental (act) values of MDA on the test sample for a reduced set of predictors: (**a**) comparison of values; (**b**) value of absolute error for test examples.

The deviations of the MDA values calculated using the reduced model from the experimental ones obtained for different samples are shown in Figure 4 and Figure 5.

**Figure 6:**
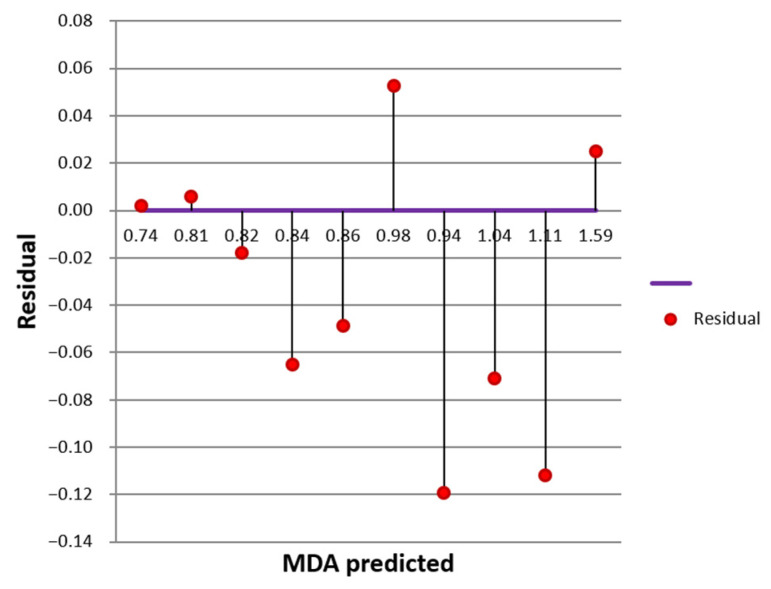
Residuals as a function of predicted MDA values for the reduced neural network model.

To further evaluate model behaviour, residuals were analysed (Figure 6). The residual distribution does not demonstrate systematic trends across the prediction range, indicating absence of systematic bias and supporting acceptable generalization behaviour of the model.

### 3.4. Primary Validation Results Using Independently Reconstructed Datasets

Because the original relational dataset contained partially replicated participant-derived relational structures, repeated cross-validation performed directly on this dataset may still partially overestimate predictive performance despite repeated random partitioning. Therefore, the following estimates are presented primarily as exploratory baseline characteristics of model behaviour rather than as rigorous leakage-free validation metrics. Within this exploratory validation framework, repeated cross-validation demonstrated relatively stable behaviour of the reduced neural network model across different dataset partitions. The mean MAE value was 0.049 ± 0.006 µmol/L, while the mean coefficient of determination was R^2^ = 0.864 ± 0.051. The empirical 95% confidence intervals ranged from 0.041 to 0.062 µmol/L for MAE and from 0.761 to 0.945 for R^2^ (Table 3).

**Figure 7:**
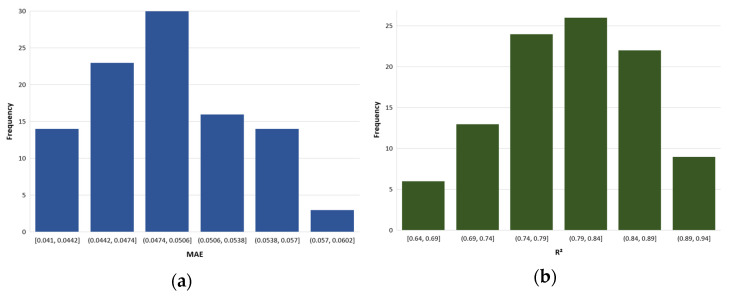
Distribution of MAE values (**a**) and R^2^ values (**b**) obtained during repeated cross-validation of the reduced neural network model.

The histogram of the model quality criterion distributions is shown in Figure 7.

These estimates are reported primarily for comparative exploratory purposes because the original relational dataset may contain partially replicated participant-derived vectors. To additionally evaluate robustness and reproducibility of the obtained machine learning results, supplementary validation experiments were performed using two independently reconstructed datasets without repeated observations.

The neural network model demonstrated relatively stable predictive behaviour across both independent datasets. For the first independent dataset, repeated 5-fold cross-validation yielded a mean R^2^ value of 0.901 ± 0.052 with a mean MAE of 0.044. For the second independent dataset, the model demonstrated a mean R^2^ value of 0.914 ± 0.046 with a mean MAE of 0.042 (Table 4).

Although some variability between folds remained present, the close agreement of predictive metrics across independently reconstructed datasets suggests that the identified nonlinear relationships are reproducible and are not solely determined by a specific partitioning configuration of the original dataset.

### 3.5. Comparative Analysis of the Effectiveness of the Proposed Model

To compare the reduced neural network model with other modern machine learning methods, we analyzed the performance of four widely used algorithms on our dataset: random forests, support vector machines (SVM), XGBoost, and LightGBM. The results for the test set are presented in Table 5.

Within the preliminary exploratory hold-out configuration based on the original relational dataset, the proposed neural network demonstrated the highest apparent coefficient of determination among the evaluated models; however, these estimates should be interpreted cautiously because the relational dataset may contain partially replicated participant-derived structures.

The neural network may better reflect complex nonlinear relationships present in the analysed dataset, whereas several alternative algorithms provide better point prediction accuracy. This difference arises because ensemble and margin-based methods optimize local error minimization, while the neural network architecture may capture broader nonlinear dependence patterns between oxidative stress markers and metal exposure parameters. Therefore, the proposed model is more suitable for analysing biological interactions rather than for individual clinical prediction.

The aim of the study is explanatory modelling rather than predictive benchmarking.

To additionally evaluate the stability of comparative model behaviour under stricter validation conditions, supplementary experiments were performed using independently reconstructed datasets without repeated observations. Under these conditions, the neural network model retained competitive predictive performance and demonstrated stable nonlinear explanatory behaviour comparable to ensemble-based machine learning methods (Table 6).

The obtained results suggest that the proposed neural network architecture does not rely solely on duplicated relational structures and remains capable of capturing reproducible nonlinear associations after removal of partially replicated relational structures. At the same time, the relatively small differences between alternative algorithms indicate that the present dataset does not support definitive conclusions regarding universal superiority of any single machine learning approach.

### 3.6. SHAP-Based Feature Importance Analysis

To improve interpretability of the neural network model, SHAP (SHapley Additive exPlanations) analysis was additionally performed.

**Figure 8:**
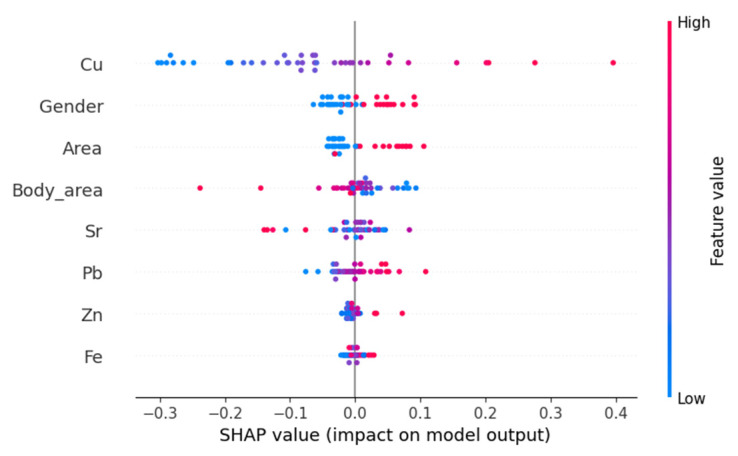
SHAP summary plot for the reduced neural network model. Predictors are ranked according to their contribution to model output variability.

For the full reduced model including demographic and environmental variables, copper concentration demonstrated the largest contribution to model output, while sex, residential area and body surface area also showed substantial influence on predicted MDA levels (Figure 8). Zinc and iron demonstrated comparatively smaller marginal contributions within the multivariate nonlinear architecture.

To further evaluate the relative contribution of trace elements independently of demographic and anthropometric variables, an additional SHAP analysis was performed using a metals-only neural network model (Table 7). In this configuration, zinc demonstrated the strongest contribution, followed by copper and iron.

These findings suggest that demographic and environmental variables explain a considerable proportion of the global variance structure in MDA levels, whereas Zn, Cu and Fe represent the principal contributors among the analysed trace elements.

Additional SHAP analyses were performed using two independently reconstructed validation datasets without repeated observations. In both datasets, zinc, copper and iron consistently remained the dominant contributors to neural network predictions, although the relative ranking between zinc and copper varied between validation scenarios (Table 8).

**Figure 9:**
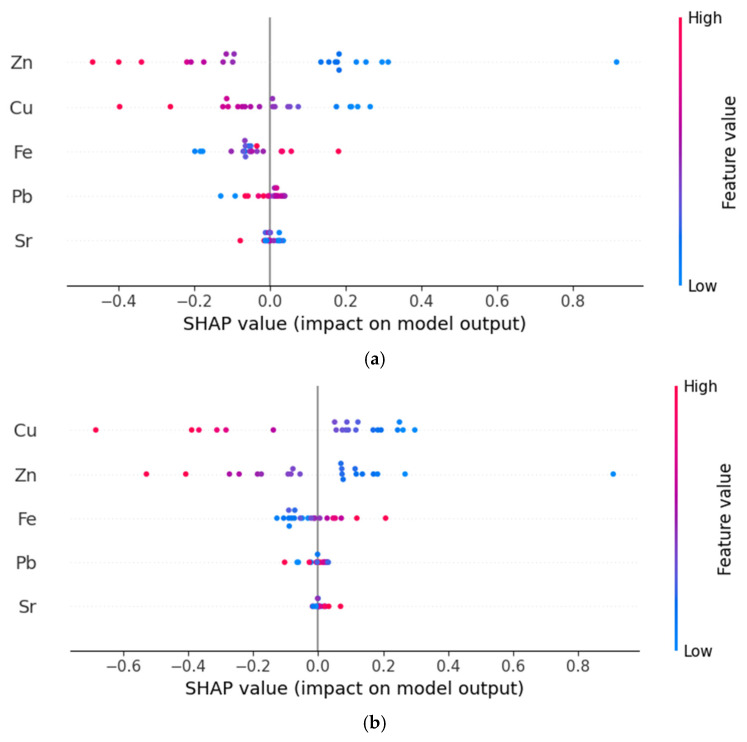
SHAP summary plot for the independently reconstructed validation datasets: (**a**) dataset 1; (**b**) dataset 2.

For the first independent dataset, zinc demonstrated the largest contribution to predicted MDA variability (mean absolute SHAP value = 0.340), followed by copper (0.199) and iron (0.075). In the second independent dataset, copper demonstrated the highest contribution (0.222), followed by zinc (0.144) and iron (0.081). Lead and strontium consistently demonstrated substantially smaller contributions in both datasets (Figure 9).

Although zinc, copper and iron consistently remained the dominant contributors across independently reconstructed datasets, the relative ranking between zinc and copper changed between validation scenarios. This behaviour indicates sensitivity of SHAP-based feature importance estimates to dataset composition and should be interpreted cautiously given the limited biological sample size.

## 4. Discussion

Analysis of the histograms of experimental MDA values and those calculated using the reduced model revealed a convergence in the distribution of values across ranges on both the training and test sets, indicating increased generalization ability of the reduced model. However, the reduced model demonstrated comparable training accuracy but substantially better generalization. Thus, the resulting model may be more suitable for exploratory description of MDA dependence patterns in blood serum.

Generalization ability represents a key property of neural network models, particularly in limited biomedical datasets. To achieve generalization, a network must be trained on a redundant dataset, since in this case the weights adapt not to individual samples but to their statistical ensembles. The generalization ability of a neural network model can be assessed using a specific measure of network complexity—the Vapnik–Chervonenkis measure, denoted as VCdim [38]. It has been established that its value depends on the number of synaptic weights connecting neurons. The larger the number of distinct weights, the higher the network complexity and, consequently, the higher the VCdim value. For adequate generalization of the input data, the ratio of the number of training samples to the VC dimension should be greater than 1 [39]. Typically, the number of training samples is known in advance and cannot be increased. Therefore, to improve the generalization ability of the network, it is necessary to reduce its complexity, i.e., the VCdim, or, using a simplified approximation for its computation, reduce the number of connections in the network.

For small biomedical datasets, generalization ability is more informative than absolute dataset size. The reliability of the proposed model is supported by the absence of divergence between training and test errors after reduction of predictors. Thus, the reduced architecture demonstrated more stable behaviour relative to dataset complexity than the initial over-parameterized configuration.

A limitation of this study is the use of a constructed dataset in which multiple relational representations were associated with the same individuals. This may introduce statistical dependencies between observations and should be interpreted with caution. The effective biological sample size remains limited. Formal statistical power calculation was not performed because the study was designed as an exploratory machine learning analysis rather than a hypothesis-testing experiment with predefined effect sizes.

The cross-sectional design does not allow causal inference. Potential confounding factors such as diet, physical activity, infections and socio-economic status were not controlled and may influence oxidative stress markers. Therefore, the results should be interpreted as associations rather than direct causal relationships. Although fully independent external validation datasets containing identical biochemical and environmental parameters are currently unavailable, the observed associations are broadly consistent with our previously published findings obtained in adolescents from the same industrial region, where elevated anthropogenic load was associated with altered serum metal profiles and increased lipid peroxidation activity [40].

An additional limitation is related to the biochemical specificity of MDA determination. Although the TBA-based assay is widely used in oxidative stress research, TBA-reactive substances may include compounds other than malondialdehyde. Consequently, the measured values should be interpreted as generalized indicators of lipid peroxidation intensity rather than fully specific markers of oxidative membrane damage. Despite these limitations, TBA-based spectrophotometric assessment of MDA remains widely used in exploratory and screening studies because it allows identification of major population-level trends and relative intergroup differences, particularly in situations involving elevated oxidative stress intensity.

Potential dietary, lifestyle and physical activity differences between urban and rural children were not formally controlled. In addition, subclinical physiological or inflammatory conditions may also influence oxidative stress markers. However, preliminary participant surveys suggested broadly similar dietary habits and lifestyle patterns between the studied groups, reducing the likelihood that major systematic differences fully explain the observed trends.

The reduced model should be interpreted as optimal for limited biomedical datasets. With larger training samples, models with a greater number of predictors may become preferable, and the present comparison reflects the effect of model capacity relative to dataset size rather than universal superiority of the reduced feature set. Supplementary comparative analysis performed on independently reconstructed datasets demonstrated that the proposed neural network model retained predictive behaviour comparable to several ensemble-based machine learning approaches after removal of partially replicated vectors. This observation supports the stability of the identified nonlinear relationships and suggests that the obtained results are not solely determined by duplicated relational structures.

Additional robustness analysis using independently reconstructed datasets without repeated observations demonstrated generally reproducible exploratory behaviour of the reduced neural network model. Validation metrics obtained on independently reconstructed datasets were slightly higher than those observed during repeated cross-validation of the original relational dataset. This behaviour may reflect reduced heterogeneity and lower intra-group variability after removal of partially replicated relational structures, resulting in a more homogeneous feature space and more stable nonlinear approximation. At the same time, repeated SHAP analyses demonstrated substantial variability in the relative ranking of zinc and copper between validation scenarios, indicating sensitivity of feature importance estimates to dataset composition and limited biological sample size. Therefore, the obtained SHAP-based rankings should be interpreted cautiously and primarily as exploratory indicators of potential nonlinear biological associations rather than as stable quantitative estimates of effect strength. Overall, the obtained results support the exploratory explanatory value of the proposed machine learning framework while emphasizing the importance of leakage-aware validation procedures and cautious interpretation of nonlinear models in limited biomedical datasets.

## 5. Conclusions

Although multiple factors can influence oxidative stress markers, the study groups were matched by age, sex and health status, and sampling conditions were standardized. Therefore, the residential environment represented the principal differing exposure factor between the analysed populations.

The study identified reproducible associations between environmental metal exposure and lipid peroxidation activity reflected by serum MDA levels in adolescents. SHAP-based feature importance analysis demonstrated that demographic, environmental and trace-element variables contributed substantially to nonlinear model behaviour, while zinc, copper and iron consistently represented the dominant contributors among the analysed metals. These findings are broadly consistent with known oxidative stress mechanisms involving transition metals and antioxidant regulation.

Primary leakage-aware validation using independently reconstructed datasets without repeated observations demonstrated reproducible exploratory behaviour of the proposed neural network model and preservation of the overall nonlinear association structure between oxidative stress markers and environmental exposure parameters. In contrast, the original 200-row relational dataset should be interpreted primarily as an exploratory modelling framework because partially replicated participant-derived relational structures may artificially inflate apparent predictive performance.

The proposed neural network approach was intended primarily for exploratory analysis of nonlinear biological interactions and model interpretability rather than for maximization of point-wise predictive accuracy. Although several alternative machine learning algorithms demonstrated lower absolute modelling error under some validation scenarios, the neural network model retained competitive explanatory capability and preserved biologically plausible association patterns.

Given the limited dataset size and exploratory study design, the obtained results should be interpreted primarily as evidence of explanatory modelling performance rather than as fully generalizable predictive accuracy. The study additionally highlights the importance of leakage-aware validation procedures and cautious interpretation of machine learning results in limited biomedical datasets with partially replicated relational structures.

The obtained results may be useful for population-level assessment of environmentally related oxidative stress and may support environmental health monitoring in pediatric populations exposed to anthropogenic trace-element imbalance. In addition, the proposed approach may contribute to risk-oriented environmental assessment strategies in which metal content in urban ecosystem components is evaluated together with potential biological effects on the human body. Such approaches may help decision-makers shift from simple concentration monitoring toward targeted health-oriented environmental risk management.

## Figures and Tables

**Table 1:** Results of the study of MDA (μmol/L) and metal (mg/L) levels in serum (M ± SD).

Research Group	MDA	Zn	Cu	Fe	Sr	Pb
Urban area						
Girls	**1.280 ± 0.125 ^1^**	0.705 ± 0.114	1.027 ± 0.391	**1.577 ± 0.333 ^1^**	0.128 ± 0.052	0.066 ± 0.017
Boys	1.011 ± 0.144	**0.979 ± 0.104 ^1^**	0.929 ± 0.168	1.113 ± 0.241	0.126 ± 0.034	0.070 ± 0.019
The whole group	**1.142 ± 0.138 ^2^**	0.812 ± 0.105	0.985 ± 0.217	1.316 ± 0.248	0.128 ± 0.048	**0.068 ± 0.017 ^2^**
Rural area						
Girls	0.858 ± 0.192	0.836 ± 0.176	0.888 ± 0.210	**1.405 ± 0.520 ^1^**	0.132 ± 0.060	0.042 ± 0.016
Boys	0.809 ± 0.176	0.945 ± 0.154	0.798 ± 0.257	0.911 ± 0.257	0.120 ± 0.053	0.039 ± 0.015
The whole group	0.847 ± 0.180	**0.941 ± 0.166 ^2^**	0.861 ± 0.218	1.304 ± 0.429	0.126 ± 0.052	0.040 ± 0.015

Note: highlighted and marked with “1” indicate statistically significant (*p* < 0.05) higher values in the intra-group comparison of boys and girls; marked with “2” (*p* < 0.01) in the inter-group comparison of urban and rural areas.

**Table 2:** Preliminary exploratory hold-out performance of the reduced neural network model using the original relational dataset.

	Training Set	Test Set
MAE error	0.034	0.051
Determination coefficient R^2^	0.921	0.945

**Table 3:** Repeated cross-validation results obtained using the original relational dataset.

Metric	Mean ± SD	95% CI
MAE (µmol/L)	0.049 ± 0.006	0.041–0.062
R^2^	0.864 ± 0.051	0.761–0.945

**Table 4:** Robustness analysis of the neural network model using independent validation datasets.

Dataset	Fold	R^2^	MAE
Independent dataset 1	1	0.941	0.038
	2	0.917	0.041
	3	0.884	0.048
	4	0.852	0.052
	5	0.909	0.043
	Mean ± SD	0.901 ± 0.052	0.044 ± 0.005
Independent dataset 2	1	0.948	0.036
	2	0.926	0.039
	3	0.901	0.041
	4	0.872	0.047
	5	0.923	0.040
	Mean ± SD	0.914 ± 0.046	0.042 ± 0.004

**Table 5:** Comparison of machine learning models for MDA modelling.

Method	MAE	R^2^
Random Forest	0.062	0.78
SVM	0.035	0.656
XGBoost	0.038	0.92
LightGBM	0.042	0.89
Neural network (proposed model)	0.051	0.945

**Table 6:** Comparative robustness analysis on independently reconstructed datasets.

Method	Independent Dataset 1 (R^2^)	Independent Dataset 2 (R^2^)
Random Forest	0.82	0.84
XGBoost	0.88	0.89
LightGBM	0.86	0.87
Neural network	0.90	0.91

**Table 7:** Feature importance for metals-only model.

Feature	Mean Absolute SHAP
Zn	0.224
Cu	0.147
Fe	0.074
Sr	0.032
Pb	0.028

**Table 8:** Feature importance for independently reconstructed validation datasets.

Feature	Independent Dataset 1	Independent Dataset 2
Zn	0.340	0.144
Cu	0.199	0.222
Fe	0.075	0.081
Pb	0.026	0.035
Sr	0.013	0.027

## Data Availability

The data presented in this study are available on request from the corresponding author due to ethical and privacy restrictions involving human participant biomedical data.
